# Subacute Combined Degeneration Secondary to Nitrous Oxide Abuse: Quantification of Use With Patient Follow-up

**DOI:** 10.7759/cureus.11041

**Published:** 2020-10-19

**Authors:** Arthur M Samia, Joseph Nenow, Donald Price

**Affiliations:** 1 Division of Neurology, Brody School of Medicine at East Carolina University, Greenville, USA; 2 Division of Neurology, Vidant Medical Center/East Carolina University, Greenville, USA

**Keywords:** subacute combined degeneration, vitamin b12, cobalamin, nitrous oxide, recreational drug use, abuse

## Abstract

Subacute combined degeneration (SCD) is caused by demyelination of spinal cord white matter secondary to vitamin B12 (cobalamin) deficiency leading to core symptoms of spastic paresis and vibratory and proprioceptive deficits. Most common causes of B12 deficiency revolve around malabsorption and pernicious anemia; however, nitrous oxide (N2O) can also indirectly cause B12 deficiency by inactivating its biologically active form. We report a case of a patient who took advantage of the unregulated N2O market and presented with signs and symptoms of SCD secondary to N2O abuse. Prior to symptom onset, the patient reported approximately 3,000g of N2O inhalation within five days prior to symptom onset in addition to daily use three weeks prior. Work up revealed laboratory and imaging abnormalities consistent with SCD, although B12 levels were normal intrinsic-factor-blocking (IFB) antibodies were present. Appropriate treatment was undertaken, and the patient was followed up at one week and one month with noticeable clinical improvements. Similarities of this patient to literature include the classic presenting symptoms of SCD and the gradual symptomatic improvement with B12 injections and N2O abstinence. This case is remarkable due to SCD occurrence after recreational N2O abuse, objective quantification of N2O intake over a specified time period to induce SCD, occurrence secondary to N2O inhalation, positive IFB antibodies, and symptomatic presentation with B12 values within normal limits. This report highlights the dangers associated with N2O abuse and moving forward awareness of this case can be referenced to aid in educating members of our communities at risk for substance abuse.

## Introduction

Subacute combined degeneration (SCD) is caused by demyelination of spinal cord white matter secondary to vitamin B12 (Cobalamin) deficiency, leading to core symptoms of spastic paresis and vibratory and proprioceptive deficits [1,2]. These symptoms result from lesions in the spinal cord’s lateral corticospinal tracts and dorsal columns, respectively [1]. Generalized symptoms can include polyneuropathy, ataxia, and psychosis [1,2]. Macrocytic anemia with hypersegmented neutrophils is a common laboratory finding associated with B12 deficiency [3,4]. The most common causes of B12 deficiency are associated with malabsorptive processes and pernicious anemia [3,4]. Other causes include the absence of a terminal ileum and insufficient dietary intake [3,4]. A rare cause of B12 deficiency can result from excess nitrous oxide (N2O) inhalation [2,4]. When inhaled in high concentrations, N2O can convert B12 from its active, bivalent form to its inactive, monovalent form, leading to indirect depletion of B12 [5].

Typically, N2O (also known as “laughing gas”) is used for patients undergoing dental procedures; however, it can also be a misused substance and carries the potential for abuse and/or addiction. Among abusers, recreational use of N2O is commonly referred to as doing “Whip-its.” When inhaled, N2O can cause an initial “rush” or “high,” followed by euphoria and numbness/tingling of the body, and occasionally dissociation from reality [6]. Users will often do multiple Whip-its in a row to achieve a persistent euphoric feeling, as its effects are transient and only last an average of 30 seconds [6]. Over the past decade, there has been a growing Whip-it epidemic, which is beginning to share the spotlight with other drugs of abuse such as opioids. The Substance Abuse and Mental Health Administration reported over 12 million N2O users in 2018 - largely popular among teens and young adults due to its ease of access and inaccurately perceived absence of significant adverse effects. In truth, N2O use has been linked not only to mild, intermittent symptoms like nausea, vomiting, dizziness, and headaches, but also more significant adverse reactions such as altered mental status, hypotension, central nervous system stimulation, apnea, anoxic brain injury, and bone-marrow suppression (long-term use) [7]. The ease of access to N2O is the reason for concern, as the market for and purchase of Whip-its is largely unregulated [8]. They can be purchased in boxes containing dozens of metal, N2O-containing canisters at grocery stores and on the Internet [8]. Here we report a case of a patient who took advantage of the unregulated N2O market and presented with SCD secondary to N2O (Whip-it) abuse.

## Case presentation

A 53-year-old man presented to the hospital with bilateral leg weakness, repeated falls, and general paresthesia with bilateral numbness in upper and lower extremities (worse in lower extremities). His past medical history was significant for uncomplicated type 2 diabetes mellitus, essential hypertension, and chronic pain. The patient reported using N2O recreationally every day, multiple times per day for one month prior to symptom onset. The patient also reported having abused N2O intermittently since adolescence. N2O Quantification: the patient inhaled one box of 25 N2O-containing steel cartridges each containing 8g (4.37L) of compressed N2O for a total of 200g (109.23L) of inhaled N2O daily for three weeks, then increased consumption to approximately 15 boxes daily containing a total of 3,000g (1,638.5L) of compressed N2O for five days prior to symptom onset.

Neurologic examination was performed upon admission. Motor examination was significant for 4/5 strength in bilateral upper and lower extremities - more pronounced in the left upper and lower extremities. Coordination examination was significant for decreased bilateral finger to nose test - more pronounced on left - and decreased bilateral heel to shin test - more pronounced on left. Reflex examination was significant for 3+ bilateral patellar reflexes with greater hyperreflexia on the left. Sensory examination was significant for impaired vibratory sense up to his knees and loss of proprioception in his bilateral lower extremities. His gait examination was significant for positive Romberg test, wide-based stance, and the patient was unable to make small steps without falling - secondary to loss of proprioception. The patient was awake, alert, and oriented to person, place, time, and circumstance throughout his admission; therefore, a mini mental examination was not deemed clinically necessary.

**Figure 1:**
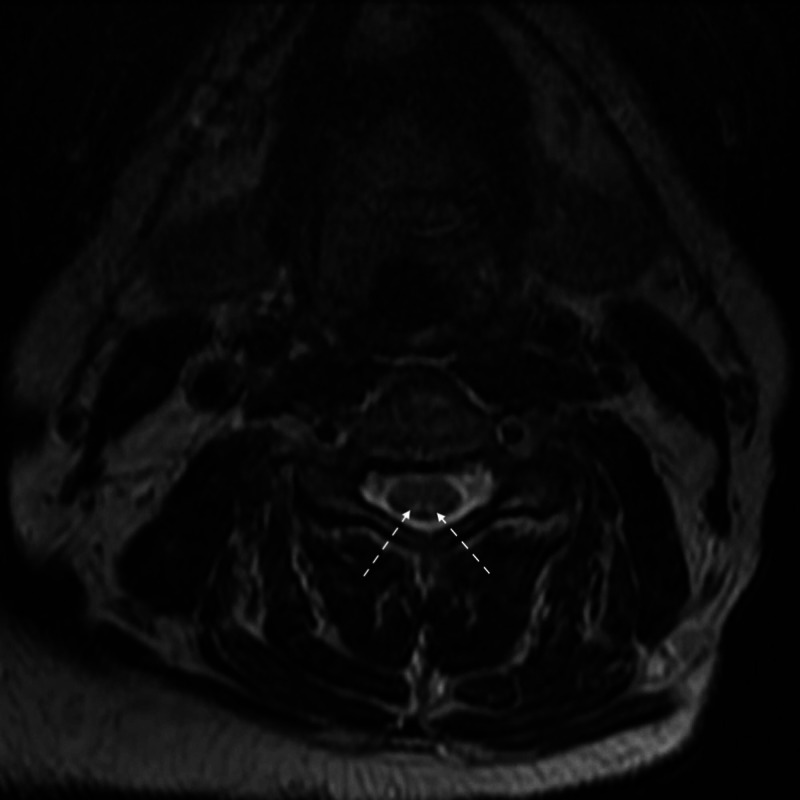
T2 axial MRI of the cervical spine showing increased signal attenuation of the dorsal columns (white arrows) MRI, magnetic resonance imaging.

Laboratory tests and diagnostic imaging modalities were performed throughout the patient’s seven-day hospital course. Work-up revealed neurologic abnormalities consistent with SCD, normal B12 levels, increased homocysteine and methylmalonic acid, positive intrinsic-factor-blocking (IFB) antibodies, and sensorimotor polyneuropathy without major demyelination features (Tables 1, 2). A peripheral blood smear was not performed. T2-weighted imaging revealed increased dorsal column signal attenuation (Figure 1).

**Table 1:** Pertinent laboratory tests taken on hospital admission with their values and reference ranges Laboratory tests: serum vitamin B12, homocysteine, methylmalonic acid, intrinsic factor blocking antibody, and vitamin E, complete blood count, and urinalysis. Two-month follow-up of serum vitamin B12 >2,000 pg/mL. *Abnormal values; ^†^clinically significant values.

Laboratory Test	Value on Admission	Reference Range
Vitamin B12	285 pg/mL^†^	232-1,245 pg/mL
Homocysteine	17.1 umol/L^*†^	<11.4 umol/L
Methylmalonic Acid	3,155 nmol/L^*†^	87-318 nmol/L
Intrinsic Factor Blocking Antibody	Positive^*†^	Negative
Hb	13.8 g/dL	13.0-17.7 g/dL
MCV	90.4 fL	79-97 fL
Nucleated RBCs	0	0-100
Vitamin E	12.1 mg/L	5.7-19.9 mg/L
Urine Ketones	Trace^*^	Negative
Glucose	121 mg/dL^*^	74-106 mg/dL

**Table 2:** Pertinent diagnostic imaging modalities and their results Electromyogram (EMG) Nerve Conduction Study, magnetic resonance imaging (MRI) of the brain, and MRI of the cervical/thoracic/lumbar spine. *Abnormal values; ^†^clinically significant values.

Diagnostic Imaging Modality	Result
EMG Test Nerve Conduction Study	Length dependent sensorimotor polyneuropathy without major demyelinating features^*†^
MRI brain	Unremarkable
MRI cervical/thoracic/lumbar spine	Multilevel disc protrusions^*^ and increased signal attenuation in the cervical cord dorsal columns^*†^

Treatment consisted of a seven-day course of daily vitamin B12 intramuscular injections at a dose of 1,000 µg, followed by once weekly injections at the same dose. At one-week office follow-up, the patient reported symptomatic improvement. There was persistence of ataxia and peripheral neuropathy associated with loss of vibratory sense and proprioception, but reflexes were 2+ throughout. At one-month office follow-up, the patient reported overall symptomatic persistence with improvement. Upon two-month telephone follow-up, the patient reported overall improvement in mobility and paresthesia; however, symptoms persisted. He had difficulty ambulating (using a cane for assistance) and performing activities of daily living due to ataxia. Symptoms were fluctuating daily, subjectively ranging from 30%-70% of baseline functionality, which was improved from almost no functionality upon admission. The patient had been supplementing his diet with 750 mg of vitamin B12 daily, as well as vitamin B6, vitamin D3, nitric oxide, and Omega-3 fatty acid since discharge. He has not been using N2O since prior to hospital admission. No further patient follow-up was reported.

## Discussion

Inhaled nitric oxide is widely used in medicine in the treatment of vaso-occlusive crisis outside of recreational use [9]. Recent case reports have shown an association between N2O anesthesia and subsequent post-anesthesia gait disorders via inactivation of B12 and downstream effects on methionine synthase and L-methylmalonyl-coA mutase [10]. In patients with SCD secondary to B12 malabsorption and deficiency, serum vitamin B12 level were found to be extremely low [10]. Conversely, nitrous oxide has been shown to induce neurologic disorders in patients with normal pre-anesthesia vitamin B12 levels. Recreational N2O has more recently been linked to SCD in a similar fashion to anesthesia.

Like the classic presentation of SCD, N2O-mediated SCD presents most commonly with complaints of limb numbness, limb weakness, and gait disturbances. In contrast to many other etiologies of SCD, N2O-mediated inactivation of B12 is not consistently tied to hematologic symptoms (e.g. macrocytic anemias) as might be expected in other causes of SCD such as pernicious anemia [11,12]. In both anesthetic and recreational use, worsened clinical manifestations are not expected to be associated with anemia, low levels of serum vitamin B12, or MRI abnormalities in the spinal cord. The age of onset and disease course are important factors in evaluating the short-term prognosis of patients with SCD [11].

When clinically evaluating and treating SCD, involuntary movements typically disappear with parenteral injections of hydroxycobalamin and abstinence of N2O, leading to gradual symptomatic improvement. This marker is an important factor when evaluating treatment efficacy. In prior studies, all patients treated with parenteral B12 showed varying degrees of clinical improvement at one-month and three-month follow-up visits [10-13]. The courses of symptom resolution could not be tied to sex, hemoglobin level, serum vitamin B12, or MRI manifestations at the time of admission or at the follow-up visits. However, both younger patients and those with shorter disease courses had better rating scores for self-perceived clinical improvement at the short-term follow-up visits [11].

Similarities of this patient to the literature include the classic presenting symptoms of SCD and the gradual symptomatic improvement with vitamin B12 injections and abstinence from N2O. This patient’s case is remarkable for a number of reasons. We report SCD occurrence after recreational N2O abuse, whereas most literature recounts incidents of SCD secondary to N2O inhalation in controlled, post-anesthetic environments [14,15]. Additionally, this is the first reported case of SCD occurrence secondary to N2O inhalation with concomitant positive intrinsic factor blocking antibodies. We also objectively quantified the patient’s N2O intake over a specified time period to induce SCD secondary to B12 inactivation - the first case reported with this data collected. This was measured to be approximately 19 kg in one month, including 15 kg over the five days preceding symptom onset. Additionally, as opposed to most cases of SCD, this patient presented with B12 values within normal limits, as inactivated B12 cannot be discerned from normal, active B12 [11]. Other laboratory values, including elevated homocysteine and methylmalonic acid levels, were consistent with all causes of SCD [11].

## Conclusions

Nitrous oxide is an uncommon cause of SCD, which can lead to permanent, debilitating neurologic damage if not appropriately and promptly recognized and treated. It should particularly be considered in younger patients and in patients with a history of substance abuse. It can be recognized through taking an accurate patient history and performing a pertinent physical examination. Laboratory tests can reveal macrocytic anemia with hypersegmented neutrophils, low or normal B12 levels, and elevated homocysteine and methylmalonic acid levels. MRI can reveal increased signal attenuation in the dorsal columns and lateral corticospinal tracts. Treatment involves cessation of the offending agent, such as N2O in our case, vitamin B12 supplementation, and physical rehabilitation.

This case report highlights the dangers associated with the mounting N2O abuse epidemic and adds to the current literature. The underregulated N2O market and its inaccurately perceived limited side effect profile makes N2O abuse a growing concern for an increase in SCD cases. Moving forward, awareness of this case and other potential cases like this can be referenced to aid in educating members of our communities at risk for substance abuse. It will also be important to further evaluate the significance of the presence of positive intrinsic factor blocking antibodies as it relates to N2O-induced SCD, as this significance is currently unexplored and unknown.
